# Refractometric Sensing with Periodic Nano-Indented Arrays: Effect of Structural Dimensions

**DOI:** 10.3390/s19040897

**Published:** 2019-02-21

**Authors:** Daniel J. Carney, Halldor G. Svavarsson, Hafez Hemmati, Alexander Fannin, Jae W. Yoon, Robert Magnusson

**Affiliations:** 1 Department of Electrical Engineering, University of Texas at Arlington, Arlington, TX 76019, USA; daniel.carney@mavs.uta.edu (D.J.C.); hafez.hemmati@mavs.uta.edu (H.H.); alexander.fannin@mavs.uta.edu (A.F.); magnusson@uta.edu (R.M.); 2 School of Science and Engineering, Reykjavik University, 101 Reykjavik, Iceland; 3 Electronics and Telecommunications Research Institute, Daejeon 34129, Korea; jaeong.yoon@gmail.com

**Keywords:** refractometer, plasmonic sensors, biosensors, nanocups, periodic structures

## Abstract

Fabrication and sensor application of a simple plasmonic structure is described in this paper. The sensor element consists of nano-patterned gold film brought about from two-dimensional periodic photoresist templates created by holographic laser interference lithography. Reflectance spectroscopy revealed that the sensor exhibits significant refractive index sensitivity. A linear relationship between shifts in plasmonic resonances and changes in the refractive index were demonstrated. The sensor has a bulk sensitivity (S_B_) of 880 nm/refractive index unit and work under normal incidence conditions. This sensitivity exceeded that of many common types of plasmonic sensors with more intricate structures. A modeled spectral response was used to study the effect of its geometrical dimensions on plasmonic behavior. A qualitative agreement between the experimental spectra and modeled ones was obtained.

## 1. Introduction

Label-free techniques for real-time monitoring of biomolecular binding events are of interest for chemical analyses and medical diagnosis. Biosensors with such ability are most commonly of optical origin, and are configured as optical waveguides, optical ring resonators, and photonic crystals, in which analytical information is acquired by monitoring changes in resonance wavelengths [1]. In a metallic sensor, surface plasmon resonance (SPR) is the operating effect. SPR can be described as the resonant oscillation of the conduction electrons at metal surfaces due to the interaction with an electromagnetic (EM) surface wave. In order to support surface plasmons (SP), the metal must have a dielectric constant with negative real and positive (small) imaginary parts. The most common metals meeting those criteria in the visible and near infrared wavelength ranges are copper, silver, and gold. In the early 1980s, Liedberg et al. applied SPR to detect a refractive index (RI) change caused by the adsorption of molecules onto a surface of a metal film [2,3]. Since then, SPR has been exploited effectively in optical biosensing technologies and has been demonstrated to hold vast potential for rapid label-free detection of chemical and biological species in numerous fields, including medical diagnostics, environmental monitoring, food safety, and security [4]. 

In general, plasmonic biosensors are composed of sensing elements that comprise metal or metal–dielectric nanostructures supporting SPs and biorecognition elements (receptors) that are able to selectively bind a target molecule (analyte). Illumination of the sensing element by light generates SPs on the nanostructures producing EM field that is highly concentrated at the surface of the nanostructures. When a solution containing analyte molecules is brought in contact with the biosensor, a capture of the analyte by a receptor immobilized on the surface of the sensing element gives rise to a change in RI in the region close to the surface. As SP characteristics are very sensitive to changes in the RI, changes in the local RI induced by the binding of analyte can be determined by measuring changes in the resonant wavelength of the light coupled to SP. Since the wave vector of a SP existing on a flat metal/dielectric interface does not match the incidence wave vector, an in-plane phase (momentum) matching scheme is employed for its excitation. Light-coupling may conveniently be brought about by subwavelength nanostructure and often the metal surface itself is periodic in order to provide the phase matching condition [5]. Depending on the nanostructure’s geometry, the SPR may be of localized (LSPR) or propagating (PSPR) nature. Examples of LSPR are the excitation of surface plasmons in nanometer-sized curved metallic structures (like gold nanoparticles and gold nanorods [6]), while surface plasmons excited in planar metal/dielectric interfaces (including sub-wavelength gratings on flat films [7]) are examples of PSPRs. While PSPR derived from thin film nanosensors typically exhibits a sensing distance on a µm scale, the (localized) sensing distance of LSPR nanosensors is on a nm scale [7]. Structures with shorter sensing lengths are more efficient for the detection of small or medium-size molecules (~<20 nm), while nanostructures with longer sensing lengths may be preferred for the detection of larger (~>20 nm) and more bulky analytes [8]. Grating coupling generating PSPR may also support LSPR invoked by field localization around apexes of the structure or rims in case of two-dimensional structures. Periodic nanostructures may therefore also support more complex modes arising from coupling of LSPR and PSPR [9]. 

In this study, we present fabrication and analyses of a class of optical biosensors composed of periodic nano-indented arrays with each indentation being a hemispherically shaped nanocup. The sensitivity of the array toward changes in RI was demonstrated using reflection spectroscopy. A shift in resonance wavelength was attained by altering the RI for the media in contact with the sensor’s surface. We suggest that the shift may be attributed to LSPR and PSPR modes coupled to evanescent cavity modes within the nanocups. A theoretical prediction of the spectral response, based on rigorous coupled-wave analysis (RCWA) was compared to the experimental observation. The novelty of the work lies in the high sensitivity of the device, which is shown to match or exceed that of many common types of plasmonic sensors obtained with more complicated processing.

## 2. Materials and Methods

### 2.1. Fabrication of Gold Nanocups

**Figure 1:**
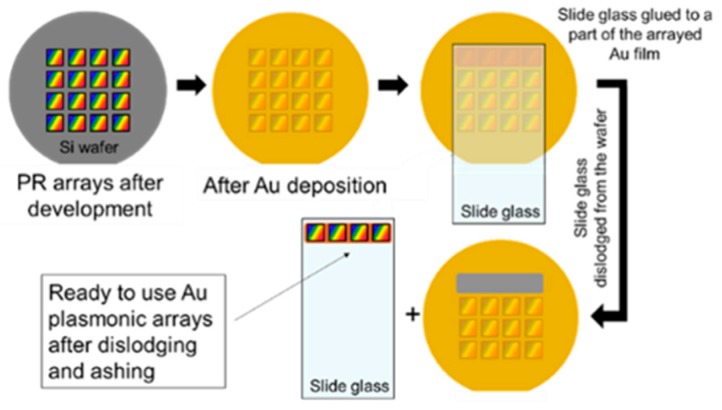
A schematic expression of the gold plasmonic sensor arrays fabrication step.

Polished silicon wafer were spin-coated with SEPR-701 positive photoresist from Shin-Etsu corporation, Tokyo, Japan, and cured on a heating plate, adjusted to 110 °C. The thickness of the photoresist (PR) film was roughly 200 nm as measured with an atomic force microscope (AFM). A continuous-wave ultra violet (λ = 266 nm) laser interference lithography system (LIL) was used to create periodic two-dimensional (2D) structures (arrays) in the photoresist (PR) through two perpendicular exposures. Sixteen patterned arrays, each being 5 × 5 mm^2^, were fabricated on a single wafer. After development, the wafer was placed on a hotplate at 190 °C for 15–20 s in order to reshape (reflow) the PR structures. By controlling the reflow temperature and reflow time, a close to perfect hemispherical shape of the nanocups was obtained [10,11]. After reflowing, a 150-nm thick Au film was sputtered on top of the PR-arrays at a rate of 20 Å/s. Subsequently, a standard 1-mm thick 3 × 1 in.^2^ glass microscope slide was glued to the gold film with epoxy adhesives as depicted schematically in Figure 1. 

After curing the epoxy, the gold film was dislodged by pulling the glass slide off. Residual PR, adhering to the gold pattern, was removed by exposure to oxygen plasma (ashing) using reactive-ion etching. A more detailed description of the fabrication process is given elsewhere [10,11]. 

### 2.2. Computed Reflection Spectra

A theoretical prediction of the spectral response for normal incidence was obtained using a commercial optical design program, RSoft DiffractMoD. The gold dispersion model given by Rakic et al. [12] was utilized to compute reflection efficiencies. A slice grid size of 0.01 μm and RCWA index resolution (in z direction) of 0.01 μm were used. In order to simplify the modeled output, all calculations were done assuming transverse magnetic (TM) light.

### 2.3. Reflectance Measurements

The optical response of the arrays was measured to determine the fabricated nanostructure’s qualities. Reflectance spectroscopy in the wavelength interval of λ = 500–900 nm was performed to monitor the plasmonic resonance of the nanocup arrays. A TM polarized light from a deuterium–tungsten lamp was used as the light source. The reflectance was measured with a USB4000 spectrometer from Ocean Optics with a detector range spanning 200–1100 nm. In order to get good average signal from the array, the spot size of the light-beam was kept rather large or close to 3 mm in diameter. Zeroth-order reflectance (R_0_) spectra were measured at normal incidence and normalized by the reflectance measured from a planar 200-nm thick Au film and the spectra for the light source. Gold thickness of the nanocups of roughly 150 nm was high enough to assure practically zero light transmittance. 

**Figure 2:**
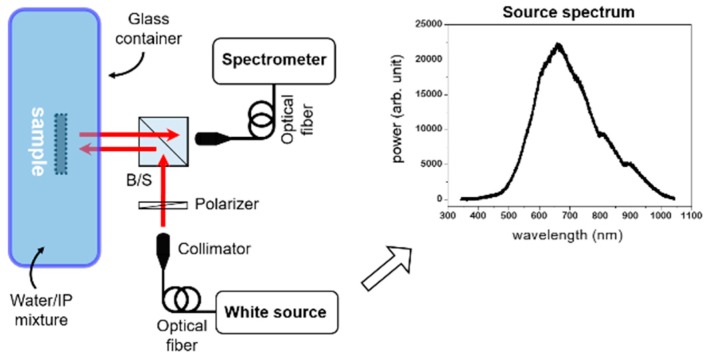
Schematic of the experimental set-up. Reflectance at normal light incidence is measured.

The measured sample was placed in the center of a square glass container (4 × 3 × 1 In.^3^) made from thin (1 mm) glass-slides (shown schematically in Figure 2). During measurement, the sample was kept immersed in a liquid solution of de-ionized (DI) water and iso-propanol (IP) in the container. We took the refractive index of DI water (n_DI_) as 1.330 and that of IP (n_IP_) as 1.374, resulting in the refractive index of the solution, n_sol_, being readily calculated as:

(1) $$n_{sol}=xn_{DI}+\left(1-x\right)n_{IP}$$

where *x* is volume ratio of DI water in the solution. IP was added to 75 mL of DI water in eight 5-mL steps. A schematic of the experimental set-up is shown in Figure 2. 

## 3. Results and Discussion

**Figure 3:**
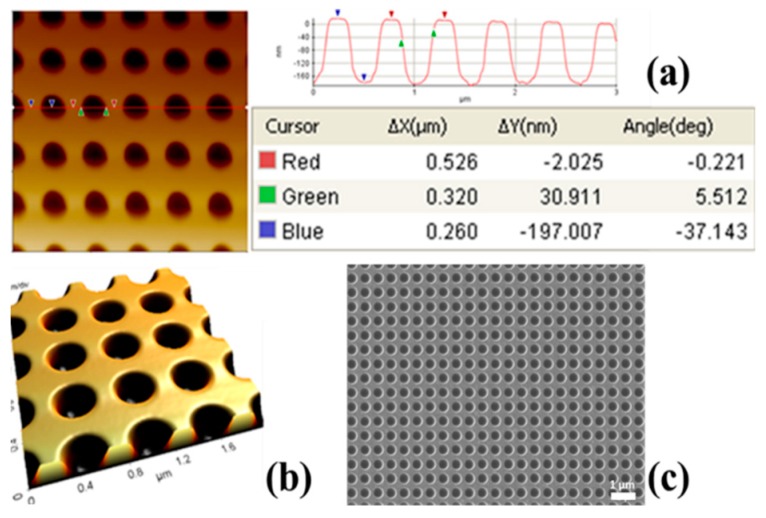
Atomic force microscope (AFM) and scanning electron microscope (SEM) measurements (**a**) two dimensional (2D AFM line scan of the array, (**b**) a 3D image generated from the AFM scan, and (**c**) an SEM image demonstrating the uniformity of the array.

A periodic array of smooth nanocups was obtained, as visualized in the atomic force microscope (AFM) and scanning electron microscope (SEM) images in Figure 3. 

For the computed spectra, the geometry of the nanocups is defined by the period (Λ), cup diameter (d), and depth (s). These values are determined using AFM measurements shown in Figure 3. 

**Figure 4:**
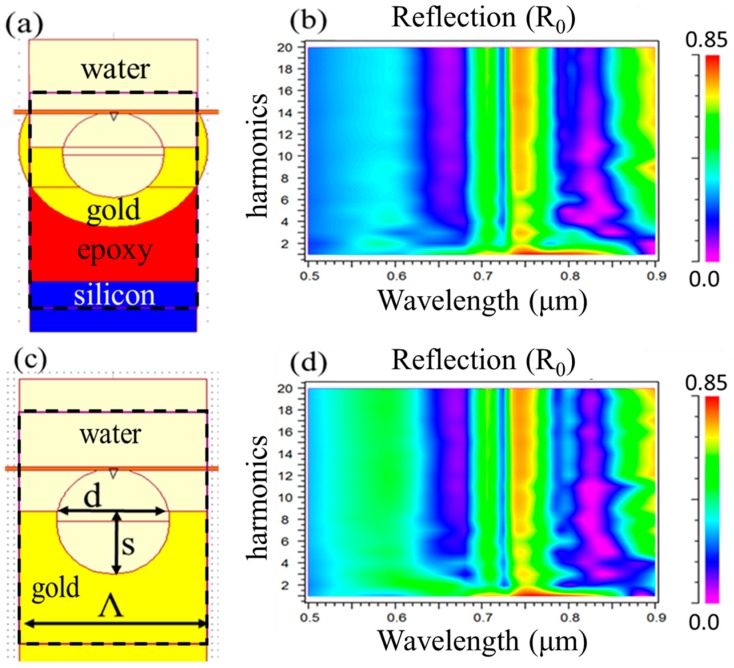
Convergence study comparing the exact model to a simplified structure. (**a**) Shows the complete model along with (**b**) its reflection spectrum plotted as a function of the number of Fourier harmonics maintained in the rigorous coupled-wave analysis (RCWA) calculation using n_epoxy_ = 1.5 and n_Si_ = 3.77. (**c**) Is a simplified bulk gold (Au) model along with its simulation results shown in (**d**). Model dimensions used are Λ = 530 nm, d = 320 nm, and s = 190 nm.

The thickness of the gold film, d_Au_, was confirmed to be sufficient to mimic that of bulk, and a convergence analysis showed that the structure can be simulated using a bulk Au substrate to reduce computation complexity as shown in Figure 4. Figure 4a,c shows the respective full and simplified models; the black dashed box outlines the simulation domain and the orange bar denotes the RCWA planewave launch point. At ~15 harmonics, both models produced similar results, and for the rest of the analysis presented here the bulk Au model was used. 

### 3.1. Theoretical Description

The response of these devices is closely linked to the physics governing transmission of light through metallic films perforated with periodic hole arrays. The theory governing these devices is reviewed by Garcia-Vidal et al. [13], where the extraordinary transmission phenomenon is explained through the coupling of diffracted surface waves to surface plasmon modes. The plasmon modes are both classical dielectric-metal interface surface plasmon polaritons (SPPs) [14] as well as effective “spoof” plasmons created by the periodic surface modulation [15]. The coupling to these plasmon modes allowed the transmission of energy through periodic metallic films—several times the skin depth in thickness—that would otherwise be optically opaque. 

**Figure 5:**
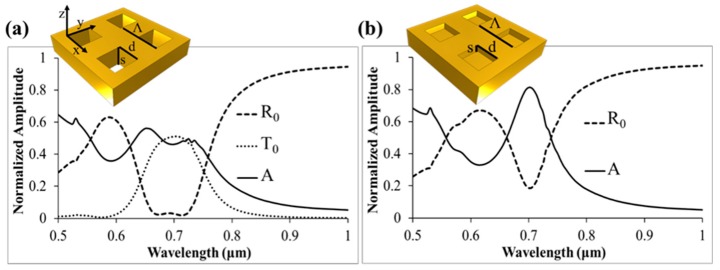
Metallic hole and nanocup array comparison. Shown is zero-order reflection (R_0_), transmission (T_0_), and absorption (A) for (**a**) a gold film with square hole array, and (**b**) a gold surface with square cup array in air. For both arrays, Λ = 530 nm, d = 273 nm, and s = 400 nm.

In Figure 5, we show the connection between a gold nanohole and nanocup array. A square period array of square holes was compared to an array of square nanocups with Λ = 530 nm, d = 273 nm for the side length, and s = 400 nm for the film thickness and hole depth. The film was assumed to be surrounded on the top and bottom by air, while the square nanocups have air on the top and solid gold substrate beneath. 

The spectrum in Figure 5a shows the typical response observed in such films, where in this case there is a strong transmission resonance around 700 nm, well into the subwavelength regime for the array periodicity and aperture size. In Figure 5b, the light is no longer permitted to transmit due to the metallic floor of the cup. Instead, it is reflected back through the evanescent cavity where additional absorption losses occur. Comparison of the two spectra shows that there is a similar resonant phenomenon in play where the transmitted energy in Figure 5a is converted to absorption losses and reflection in Figure 5b at λ = 700 nm. 

**Figure 6:**
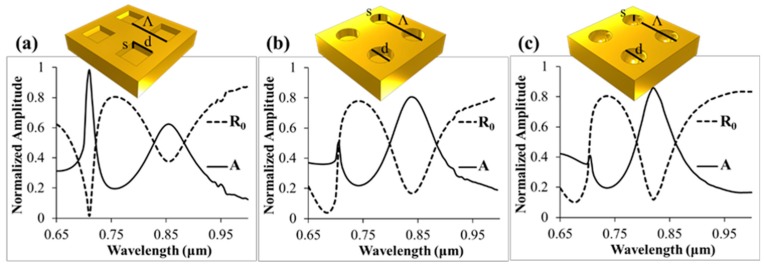
Comparison of nanocup geometry. Reflection and absorption amplitudes are show for (**a**) square, (**b**) cylindrical, and (**c**) hemispherical cup arrays in water. The dimensions are matched such that the cutoff wavelengths of the apertures are the same. Λ = 530 nm and s = 190 nm for all three arrays. For the square array, d = 273 nm, for the cylindrical and spherical array, d = 320 nm.

In Figure 6 we compare the absorption and reflection spectra for square, cylindrical, and spherical cup geometries with Λ = 530 nm and cup depth s = 190 nm. The cups were assumed to be immersed in water with n = 1.33. The cylindrical and spherical cups had the same diameter, d = 320 nm, while the square cup had a side d = 273 nm. The cavity opening dimensions were chosen to create circular and square waveguides with matching cutoff frequencies. 

The rationale for this matching relates to the derivation of the effective plasma frequency, ω_p_, for a periodically modulated metallic surface as described in [15], where ω_p_ is equal to the cutoff frequency of the waveguide defined by the aperture shape. Adjusting the dimensions to match the cutoff frequencies resulted in similar spectral responses, which indicated a strong connection between the resonance and the coupling to cavity modes. Comparing the square and cylindrical spectra to the spherical cup spectrum, made it apparent that similar resonant responses were present in all three cases. Differences between the spectra can be attributed to the differences in the shape of, and associated coupling to the fundamental modes for a square and circular waveguide, and to the tapered waveguide shape of the spherical geometry in Figure 6c. Sensitivity calculations (not shown here) executed for the square, cylinder, and spherical shapes showed no significant difference in the sensitivity of the cavity resonance.

### 3.2. Measured Reflectance Spectra

**Figure 7:**
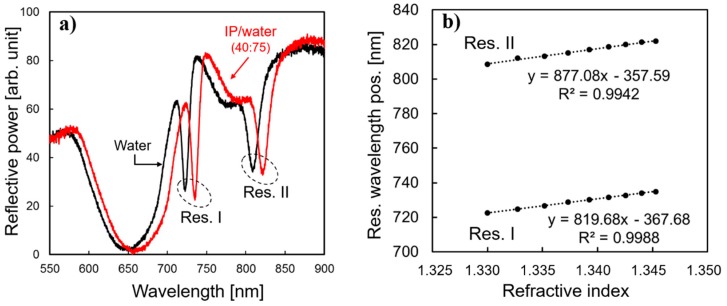
(**a**) Experimental reflectance spectra for a nanocup array in water (black line) and in 40:75 iso-propanol (IP)/water media (red line). For the sake of clarity, only spectral curves for the lowest and highest ratio of IP are shown. (**b**) Positions of the two resonance absorptions (Res. I and Res. II in (**a**)) as a function of the media’s refractive index for different IP/water ratios.

The wave vector of the excitation beam is wavelength dependent and it only matches with the propagation constant of the surface plasmon waves and the evanescent cavity modes at particular wavelengths. At resonance, the incidence light is phase matched to the surface modes and there is energy transfer between the light and surface cavities. Consequently, the intensity of the reflected light is attenuated and creates resonant reflection dips in the spectrum as seen in Figure 7. 

Resonance features of TM modes are apparent as drops in the R_0_ intensity occur. Notice, however, that for a periodic and symmetrical 2D structure and normal light incidence (as here), the spectra obtained from either a transverse electric (TE) or TM polarized light-source should be the same since the incoming light would experience the same environment upon a 90° in-plane rotation of the array. 

The most widely used performance characteristic of a plasmonic sensor is the ability to detect changes in the RI [9]. The RI of water is close to being a constant of 1.330 over the wavelength range measured, but increases upon mixing with IP. The IP was gradually added in steps of 5 mL to a 75 mL volume of DI-water up to a volume ratio 40:75, thus increasing the RI value from 1.330 to 1.345. Figure 7a shows measured reflectance spectra of a nanocup array immersed in liquid media (Λ = 530 nm, cup diameter = 320 nm, and depth = 190 nm) for TM polarized light under normal incidence in the wavelength range 550–900 nm. Two sharp anti-reflection points were observed, at roughly 722 nm (Res. I) and 809 nm (Res. II). Upon adding 40 ml of IP to the 75 mL of water, a substantial red shift of the resonance dips was visualized (red line). For clarity, only spectra for the pure water (black line) and the highest IP/water ratio (red line) are shown in Figure 7. Figure 7b shows the positions of the two resonance dips from Figure 7a as a function of the media’s refractive index for various IP/water ratios. A linear fitting of the data points had regression coefficients (R^2^) close to unity (0.9988 for Res. I and 0.9942 for Res. II), demonstrating a firm linear relation between the resonance shift and RI of the media. The wavelength shifts of the resonances per RI-unit (nm/RIU) measured as 820 nm and 877 nm for Res. I and Res. II, respectively. In addition, the appearance of two resonance dips (instead of just one) comprised further sensitivity on their own. The higher RIU sensitivity at Res. II compared to Res. I, is mainly explained on the basis of the linear nature of Maxwell’s equations, that resonant phenomenon at longer wavelengths will have longer resonant shifts. Comparing the ratios of the center wavelength to the wavelength shift at Res. I and Res. II, we got 722/820 = 0.88 and 809/877 = 0.92, respectively. Although the ratio at Res. II was still slightly higher, the remaining 5% difference can be explained by the characteristics of the two resonances. Res. I is SPP related to the slight angular incidence, while Res. II is strongly connected to the cavity modes of the nanocups. 

The resonance shifts observed (820 and 877 nm/RIU) were higher than typically reported for LSP sensors, and among the highest values for LSP–PSP coupled mode sensors [9], and yet with simpler device fabrication. Monteiro et al, for example, managed to get a bulk sensitivity of 455 nm/RIU for a SPR sensor, based on arrays of periodically (Λ = 465 nm) perforated gold film on a glass substrate [16]. Joshi et al [17,18] reported SB of up to 647 nm/RIU for a LSR biosensor based on gold nanoprisms. Substantially higher values of SB have been reported for delocalized modes compared to localized modes. A biosensor composed of goldfilm with nanohole arrays on a hybrid substrate (fused silica covered with silicon nitride film) with refractive sensitivity of 671 nm/RIU has been reported [19]. The holes had sharp sidewalls (rectangular cross-section), diameter of 200 nm, and periodicity of 600 nm. A recent paper on high-performance refractive index sensor, based on guided-mode resonance (referred to as Fano-resonance in the paper) reported a sensitivity of 452 nm/RIU [20]. 

### 3.3. Wave Propagation 

**Figure 8:**
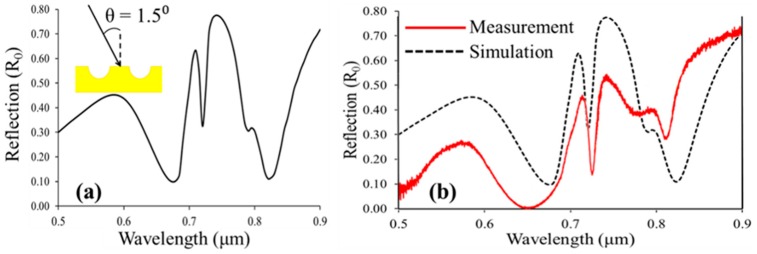
Off-normal incidence and curved wavefront simulations for nanocups immersed in water. (**a**) Transverse magnetic (TM) polarized planewave response with angle of incidence θ = 1.5°. (**b**) Averaged spectrum from 1°–2° (black line) and measured spectrum (red line).

In the modeling shown in Figure 5 and Figure 6, the wavefront of the incoming light was assumed to consist of normally incident infinite parallel planewaves. The spectral response for the simulated devices was markedly different from experiment. Further comparisons of the measured data were made by averaging the results from angular sweep simulations to give an indication of what the expected spectrum might be from a curved (Gaussian) wavefront. A qualitative fit to the experimental results was observed, as shown in Figure 8. 

In order to model alignment errors, the angle θ, defined in the modeling software as the inclination from the x-z plane, was altered to determine its impact as shown in Figure 8. Two sharp anti-reflections dips were seen in Figure 8a where θ = 1.5°. The first dip at 720 nm corresponded to SPP resonance and was roughly aligned with the first dip in the measured spectrum. The second strong peak at 825 nm remained as well and corresponded to the second resonant point in the measured data. At this angle we also noted the suggestion of a shoulder point just before 800 nm that also appeared in the measured results. In Figure 8b we show an average spectrum derived from planewaves incident from θ = 1° to 2° that begins to simulate a slightly off normal incidence curved wavefront, becoming more closely aligned with the measured results.

## 4. Conclusions

In this study, we investigated the use of simple plasmonic nanostructure as a sensitive refractive index sensor to demonstrate reflectance spectroscopy. The sensor element was made from a gold surface modulated with periodic arrays of hemispherical nano-indentation using a fabrication method developed earlier by the author’s [10,11]. A sensitivity of roughly 9 × 10^2^ nm/RIU was observed, which is comparable to or higher than reported for most plasmonic sensors, fabricated in more complicated ways. Supplemental computational modeling indicated that the curvature of the nano-indentations plays a relatively small role in the overall shape of the resonance and provides for wide tolerances useful in fabrication. The plasmonic nanostructures hold potential as optical label-free biosensors for the detection of chemical and biological substances.
